# Glucose‐loading reduces bone remodeling in women and osteoblast function in vitro

**DOI:** 10.14814/phy2.12700

**Published:** 2016-02-04

**Authors:** Itamar Levinger, Ego Seeman, George Jerums, Glenn K. McConell, Mark S. Rybchyn, Samantha Cassar, Elizabeth Byrnes, Steve Selig, Rebecca S. Mason, Peter R. Ebeling, Tara C. Brennan‐Speranza

**Affiliations:** ^1^ Clinical Exercise Science ProgramInstitute of Sport, Exercise and Active Living (ISEAL)Victoria UniversityMelbourneAustralia; ^2^ Department of EndocrinologyAustin HealthUniversity of MelbourneMelbourneAustralia; ^3^ College of Health and BiomedicineVictoria UniversityMelbourneAustralia; ^4^ Department of PhysiologyBosch Institute for Medical ResearchUniversity of SydneySydneyAustralia; ^5^ PathWest QEII Medical CentrePerthAustralia; ^6^ School of Exercise & Nutrition SciencesDeakin UniversityMelbourneAustralia; ^7^ Department of MedicineSchool of Clinical SciencesFaculty of Medicine, Nursing and Health SciencesMonash UniversityMelbourneAustralia

**Keywords:** Bone remodeling, exercise, glycemic control, in vitro, in vivo, osteoblasts function

## Abstract

Aging is associated with a reduction in osteoblast life span and the volume of bone formed by each basic multicellular unit. Each time bone is resorbed, less is deposited producing microstructural deterioration. Aging is also associated with insulin resistance and hyperglycemia, either of which may cause, or be the result of, a decline in undercarboxylated osteocalcin (ucOC), a protein produced by osteoblasts that increases insulin sensitivity. We examined whether glucose‐loading reduces bone remodeling and ucOC in vivo and osteoblast function in vitro, and so compromises bone formation. We administered an oral glucose tolerance test (OGTT) to 18 pre and postmenopausal, nondiabetic women at rest and following exercise and measured serum levels of bone remodeling markers (BRMs) and ucOC. We also assessed whether increasing glucose concentrations with or without insulin reduced survival and activity of cultured human osteoblasts. Glucose‐loading at rest and following exercise reduced BRMs in pre and postmenopausal women and reduced ucOC in postmenopausal women. Higher glucose correlated negatively, whereas insulin correlated positively, with baseline BRMs and ucOC. The increase in serum glucose following resting OGTT was associated with the reduction in bone formation markers. D‐glucose (>10 mmol L^−1^) increased osteoblast apoptosis, reduced cell activity and osteocalcin expression compared with 5 mmol L^−1^. Insulin had a protective effect on these parameters. Collagen expression in vitro was not affected in this time course. In conclusion, glucose exposure reduces BRMs in women and exercise failed to attenuate this suppression effect. The suppressive effect of glucose on BRMs may be due to impaired osteoblast work and longevity. Whether glucose influences material composition and microstructure remains to be determined.

## Introduction

Skeletal health is maintained throughout life by the cellular machinery of bone remodeling, the final common pathway mediating the effects of all genetic and environmental factors on the material composition, microstructure, and strength of bone (Parfitt 2008). Around midlife bone remodeling becomes unbalanced as the volume of bone formed by each basic multicellular unit (BMU) is less than was resorbed (Lips et al. 1978). Each time bone is remodeled, a small volume of bone is lost, structural deterioration follows and increases bone fragility.

Thus, the age‐related reduction in the volume of bone formed by each BMU plays a central role in the pathogenesis of bone fragility. Aging is also associated with insulin resistance. The insulin resistance and any existing hyperglycemia may each reduce the volume of bone formed by osteoblasts of the BMU and reduce the rate of bone remodeling. For example, bone remodeling markers (BRMs) are suppressed after a meal or following a glucose‐load in healthy women and in women with insulin resistance (Clowes et al. 2002; Schwetz et al. 2014). In obese subjects, higher serum glucose is associated with lower levels of BRMs (Iglesias et al. 2011).

The suppressive effect of a glucose‐load is likely to be related to the high circulating glucose levels and not the accompanying higher insulin levels as insulin is anabolic and is capable of promoting osteoblastic proliferation and differentiation as well as inducing increased markers of bone formation like alkaline phosphatase (ALP) and osteocalcin (OC) (Yang et al. 2010). Although the reduction in bone formation produced by hyperglycemia may produce structural decay, any slowing of remodeling produced by hyperglycemia may attenuate bone loss and structural deterioration accounting for the finding of normal or high bone mineral density (BMD) in women with diabetes. However, the slowing of remodeling may compromise bone's ‘qualities’; the brittleness of the unremodeled bone matrix is increased by increasing collagen cross‐linking and increasing the completeness of tissue mineralization which may explain the increased fracture risk in women with type 2 diabetes despite normal or higher BMD (Schwartz et al. 2011; Leslie et al. 2012).

Undercarboxylated osteocalcin (ucOC) participates in glucose homeostasis in mice and humans (Lee et al. 2007; Iglesias et al. 2011; Levinger et al. 2011; Brennan‐Speranza et al. 2012; Schwetz et al. 2014). Osteocalcin (OC), a protein synthesized exclusively by osteoblasts, undergoes posttranslational carboxylation but not all of the OC is carboxylated; some remains undercarboxylated(Pittas et al. 2009) and this osteoblast product contributes to the regulation of insulin secretion and sensitivity in both mice and human subjects (Lee et al. 2007; Ferron et al. 2008, 2012; Iglesias et al. 2011; Levinger et al. 2011; Brennan‐Speranza et al. 2012; Schwetz et al. 2014).

Obese subjects and patients with type 2 diabetes have reduced circulating ucOC, which compromises insulin sensitivity resulting in higher glucose levels (Fernandez‐Real et al. 2009; Kanazawa et al. 2009, 2011; Brennan‐Speranza and Conigrave 2015). However, it is plausible that this is bidirectional; low ucOC cause high glucose, and high glucose reduces ucOC (Paldanius et al. 2012). Exercise does the reverse – it increases insulin sensitivity by improving muscle glucose uptake and, partly, by increasing ucOC (Levinger et al. 2011, 2014). OC, ucOC and *β*‐isomerized C‐terminal telopeptides (*β*‐CTX) increase after acute exercise (Guillemant et al. 2004; Scott et al. 2010; Levinger et al. 2011, 2014). Whether exercise prevents the suppressive effect of a glucose‐load on BRMs in women is not clear.

Thus, prospective studies are needed to determine whether a glucose‐load reduces circulating ucOC levels in women. Moreover, as BRMs are affected by many confounders in vivo, in vitro studies are needed to identify the direct role of glucose and insulin on osteoblasts. We hypothesized that (1) at rest, a glucose‐load will suppress BRMs, and (2), that exercise prior to glucose‐load increases BRMs thereby attenuating the suppressive effect of the glucose‐load, (3) that there will be direct effects of glucose and insulin on cultured primary human osteoblast survival and function in vitro*;* high glucose will stimulate apoptosis and reduce osteoblast viability and ALP activity, and expression of the bone formation markers osteocalcin and type 1 collagen. Furthermore, we hypothesized that insulin treatment will prevent the negative effects of high glucose on osteoblasts in vitro.

## Materials and Methods

### In vivo study

#### Participants

Premenopausal women (*n* = 8, age = 36.1 ± 2.7 years, BMI = 25.5 ± 0.8) were eligible for the study if they were between 20 and 50 years of age and had regular menstrual cycles (Table 1). Postmenopausal women (*n* = 10, age = 62.8 ± 2.6 years, BMI = 28.3 ± 1.3 kg m^−2^) were eligible if they experienced >12 months of amenorrhea as a result of a natural menopause. Women who were perimenopausal, with musculoskeletal or other conditions that prevented daily activity and symptomatic or uncontrolled metabolic or cardiovascular disease and those taking warfarin or vitamin K supplementation, antihyperglycemic medications were excluded. Each participant was given written and verbal explanations about the study before signing an informed consent form. The study protocol was approved by the Human Research Ethics Committee, Victoria University.

**Table 1:** Group characteristics

	Premenopause (*n* = 8)	Postmenopause (*n* = 10)	*P*‐value
Age (years)	36.1 ± 2.7	62.8 ± 2.6	<0.001
Height (cm)	162.7 ± 1.8	161.2 ± 1.6	0.54
Body mass (kg)	66.4 ± 1.9	73.6 ± 3.4	0.10
BMI (kg m^−2^)	25.5 ± 0.8	28.3 ± 1.3	0.12
Glucose (mmol L^−1^)	4.4 ± 0.3	4.9 ± 1.0	0.15
HbA1c (%)	5.3 ± 1.0	5.5 ± 1.0	0.16
VO_2peak_ (mL kg^−1^ min^−1^)	24.2 ± 2.1	19.7 ± 1.6	0.09
tOC (ng mL^−1^)	19.8 ± 2.0	28.2 ± 2.0	0.01
UcOC (ng mL^−1^)	9.9 ± 1.0	13.9 ± 1.4	0.04
P1NP (*μ *L^−1^)	43.4 ± 4.4	67.2 ± 7.6	0.02
*β*‐CTX (*μ *L^−1^)	368.9 ± 47.1	429.3 ± 40.1	0.34

BMI, body mass index; HbA1c, glycosylated hemoglobin; tOC, total osteocalcin; ucOC, undercarboxylated osteocalcin; P1NP, procollagen type 1 N‐terminal propeptide; *β*‐CTx, *β*‐isomerized C‐terminal telopeptides.

#### Study protocol

The study was a randomized‐control, crossover design. Participants underwent an initial assessment including anthropometric measurements, assessment of peak aerobic power (VO_2peak_) and a fasting blood test. Following the completion of the baseline assessments, participants were randomly assigned to two sessions of an oral glucose tolerance test (OGTT) following rest (control trial) and following an acute bout of aerobic exercise. The two sessions were performed approximately 1 week apart.

Weight was measured using a calibrated scale (TANITA, Tanita Corporation, Tokyo, Japan). Height was measured with an electronic stadiometer (Proscale, Accurate Technology Inc., Fletcher, NC). Participants were required to wear a 12‐lead electrocardiogram (ECG; Mortara, X‐Scribe II, Milwaukee, WI) during a symptom‐limited graded exercise test, performed on a Cybex MET 100 cycle ergometer (Cybex Metabolic Systems, Ronkinkoma, NY) as described previously (Levinger et al. 2007, 2014). The graded exercise test was terminated when a rating of perceived exertion of ‘very hard’ (Borg scale = 17) was achieved. VO_2_ for each 15‐s interval was measured by gas analysis (Medgraphics, Cardio2 and CPX/D System with Breezeex Software, 142090‐001, Revia, MN), which was calibrated before each test. A blood sample was collected following an overnight fast. Blood was analyzed at Austin Health pathology (Melbourne, Australia) using standard hospital assay protocols for glucose, HbA1c and insulin.

Participants performed a standard 75 g of OGTT following an overnight fast. Venous blood samples were collected at baseline and 120 min after the glucose load, centrifuged and immediately stored at −80°C. Serum was analyzed for glucose, insulin, and BRMs. In the second trial, acute aerobic exercise was performed on a cycle ergometer for 30 min at an intensity corresponding to 70–75% of VO_2peak_. The exercise intensity was adjusted every 5 min to maintain a heart rate range in the desired intensity. Participants recovered for 60 min before undertaking a standard 75 g OGTT.

Blood samples were obtained immediately postexercise and at 30 and 60 min post exercise and 120 min after the glucose load. Total serum OC was measured using an automated immunoassay (Elecsys 170; Roche Diagnostics). This assay has a sensitivity of 0.5 *μ*g L^−1^, with an intra‐assay precision of 1.3%. Serum ucOC was measured by the same immunoassay after adsorption of carboxylated OC on 5 mg/mL hydroxyl‐apatite slurry, following the method described by Gundberg et al. (1998). The ucOC values on these samples of 6.0–35.6 *μ*g L^−1^ and % unbound osteocalcin between 38.1 and 51.4% are in keeping with the original assay validation. The interassay CV for total OC is 8.3% and the interassay CV for ucOC is 5.7%. tOC and ucOC assays were performed at PathWest QEII Medical Centre, Perth. Insulin, *β*‐isomerized C‐terminal telopeptides (*β*‐CTx, a bone resorption marker) and procollagen 1 N‐terminal propeptide (P1NP, a bone formation marker) were analyzed at Austin pathology, Melbourne using a Roche Hitachi Cobas e602 immunoassay analyzer, according to the manufacturer's guidelines.

### In vitro study: assays of cultured human osteoblasts (HOBs)

#### Cell culture

Primary human osteoblasts (HOBs) were grown from the minced trabecular ends of fetal long bone in accordance with the National Health and Medical Research Council guidelines and with the approval of the University of Sydney Human Ethics Committee (approval number:2012/043), as previously characterized and described (Slater et al. 1994; Sivagurunathan et al. 2005; Brennan et al. 2009; Rybchyn et al. 2011). These cells express ALP, osteocalcin, OPG, RANKL and sclerostin in cultures (Slater et al. 1994; Sivagurunathan et al. 2005; Brennan et al. 2009; Rybchyn et al. 2011). The study was conducted using HOBs from several different donors. Each experiment was conducted using HOBs from at least two different donors. HOBs were routinely maintained in DMEM containing 10% (v/v) FCS supplemented with 150 μmol L^−1^ L‐ascorbic acid 2‐phosphate and *β*‐glycerophosphate (10% DMEM). HOBs were seeded in 10% DMEM at 1 × 10^4^ cells per well and allowed to attach overnight in 96 well plates for Cell Titer Blue, ALP, Caspase‐3 and BCA assays and at 1 × 10^6^ cells per well in six well plates for RNA extraction followed by PCR. HOBs were equilibrated in glucose‐free, serum‐free Martinez buffer solution for 90 min prior to the addition of media containing 0, 2.5, 5, 10, and 20 mmol L^−1^ D‐Glucose with or without insulin (50 *μ*U mL^−1^, physiological levels observed in postmenopausal women in this study following OGTT) for 2 h, which was specifically chosen to replicate the time frame of the OGTT performed in this study in humans. All in vitro assays were performed on three separate occasions, each in quintuplicates. Data presented are combined from each assay.

#### Cell viability

Following treatment, HOB viability was assessed using Cell Titer Blue (Promega, Wisconsin) performed according to the manufacturer's instructions.

Alkaline phosphatase (ALP) activity is used as a marker of mature, bone‐forming osteoblasts (Szulc and Delmas 2008). HOBs were lysed in PBS containing 0.1% (v/v) TX‐100 followed by the addition of p‐Nitrophenyl Phosphate (p‐NPP) to a final concentration of 1 mg mL^−1^ in 0.1 mol L^−1^ Glycine pH 9·5. The formation of the colored product was monitored at 405 nm and the amount (units; U) of ALP was interpreted from a standard curve of recombinant ALP (Promega) incubated under the same conditions.

Apoptosis was assessed by measuring activated caspase‐3 activity, using the fluorogenic Caspase‐3 substrate Ac(N‐acetyle)‐DEVD‐AFC (7‐amino‐4‐trifluoromethylcoumarin) (Becton Dickinson, New Jersey) used according to the manufacturer's instructions. Data were corrected for total cell protein using the bicinchoninic acid assay (Pierce) on cells plated at the same density on the same plate that had undergone the same treatment.

Osteocalcin and type 1 collagen RNA expression was assessed by reverse transcription PCR. RNA was extracted from HOBs plated in 6‐well plates using a QIAGEN RNeasy Mini extraction kit according to the manufacturer's instructions. Total RNA (2 *μ*g) was converted to cDNA using the Bioline SensiFAST^™^ cDNA Synthesis kit. PCR was performed using the

iTaq^™^ Universal SYBR Green Supermix kit according to manufacturer's instructions and the following primers designed specifically for this study: osteocalcin Fwd:5′ATGAGAGCCCTCACACTCCTC and Rev:5′CGGGCCGTAGAAGCGCCGAT, and col1a1 Fwd:5′CTGACCTTCCTGCGCCTGAT and Rev: 5′ GTCTGGGGCACCAACGTCCA. Human b2‐microglubulin (hB2M) was used to normalize the expression of osteocalcin and collagen. hB2M primers were as follows: Fwd:5′GCCTTAGCTGTGCTCGCGCT and Rev: 5′ACCTGAATCTTTGGAGTACGCT. 5 mmol L^−1^ glucose‐treated cells was chosen as the calibrator sample.

### Statistical analyses

A repeated‐measures analysis of variance (ANOVA) with a post hoc analysis, LSD correction, was used to identify differences between time points in the in vivo study. Differences between glucose concentrations in the in vitro study were analyzed with a multivariate analysis with LSD correction. In addition, differences between glucose alone and glucose plus insulin for each pair HOBs at a specific glucose concentration were analyzed with paired *t* test. Multilinear regression model with age, BMI, serum glucose, and insulin levels following resting and exercise OGTTs were used to determine association with BRMs. All data are reported as mean ± standard error of mean (SEM) and all statistical analyses were conducted at the 95% level of significance (*P* ≤ 0.05).

## Results

### In vivo

#### Glucose and insulin levels following resting and exercise OGTTs

**Figure 1:**
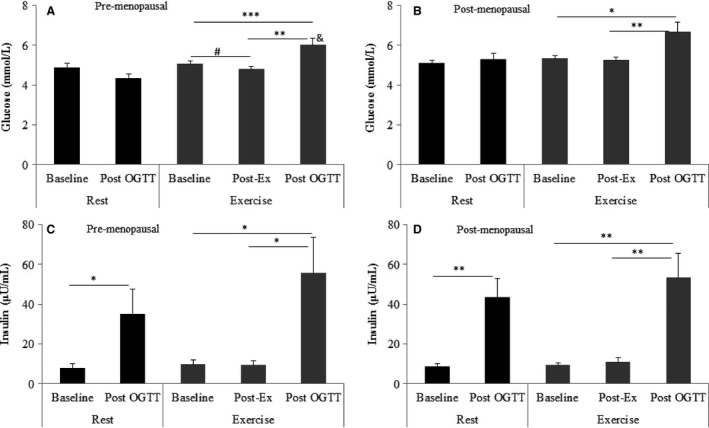
The effects of glucose load and acute exercise on serum glucose and insulin levels in pre (A and C) and post (B and D)menopausal women. *indicates *P* < 0.05

Serum glucose returned to baseline in pre and postmenopausal women 2 h following the OGTT at rest (Fig. 1A and B). Serum glucose was 20–25% higher after the exercise OGTT compared with the resting OGTT (*P* < 0.05; Fig. 1A and B). Exercise had no effect on serum insulin in pre or postmenopausal women (Fig. 1C and D). However, serum insulin was elevated four to sixfold (*P* < 0.05) in both groups after OGTT in resting and exercise experiments (Fig. 1C and D).

#### Bone remodeling markers and ucOC levels following exercise and glucose‐load

**Figure 2:**
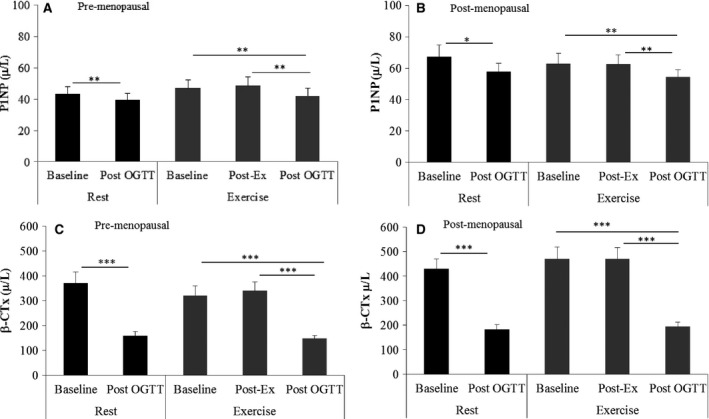
The effects of glucose load and acute exercise on serum P1NP and β‐CTX in pre (A and C) and post (B and D)‐menopausal women. *P ≤ 0.05, **P ≤ 0.01, ***P ≤ 0.001.

In the resting trial, glucose‐load suppressed P1NP (10–13%, *P* < 0.05) and *β*‐CTX by two‐fourfold (*P* < 0.001) in pre (Fig. 2A and C) and postmenopausal women (Fig. 2B and D). Exercise OGTT had no effect on P1NP and *β*‐CTX in either population (Fig. 2A–D). Similar to the resting trial, both markers were suppressed two hours post glucose load that commenced after exercise in pre and postmenopausal women (*P* < 0.01, Fig. 2A–D).

**Figure 3:**
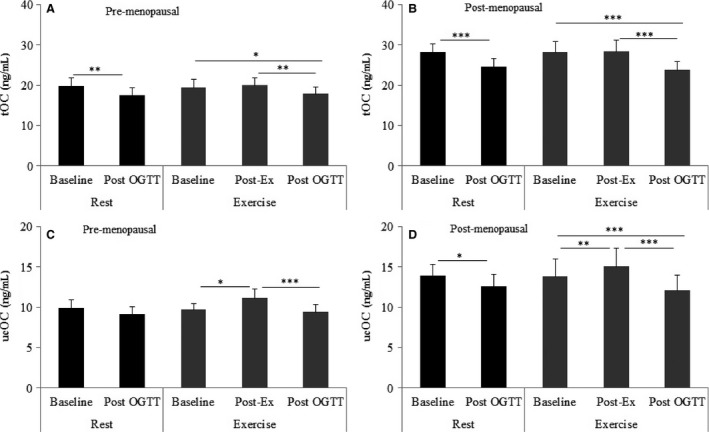
The effects of glucose‐load and acute exercise on serum tOC (total osteocalcin) and ucOC (undercarboxylated osteocalcin) in pre (A and C) and post (B and D)‐ menopausal women. *P ≤ 0.05, **P ≤ 0.01, ***P ≤ 0.001.

Figure 3 shows the effects of glucose‐load and exercise on tOC and ucOC. In the resting trial, tOC was suppressed 2 h post‐OGTT in pre (~12%, *P* = 0.001, Fig. 3A) and postmenopausal women (~13%, *P* = 0.001, Fig. 3B). Compared to baseline levels, ucOC was suppressed, but not statistically so, following resting OGTT in premenopausal women (−8%, *P* = 0.16, Fig. 3C) and in postmenopausal women (~12%, *P* = 0.03, Fig. 3D).

Exercise had no effect on tOC in pre or postmenopausal women (Fig. 3A and B). In contrast, exercise increased ucOC in pre (~14%, *P* = 0.039, Fig. 3C) and postmenopausal women (~9%, *P* = 0.008, Fig. 3D).

In postmenopausal women, tOC and ucOC were suppressed 2 h after the exercise OGTT (8–20%, *P* < 0.05), with no differences between the resting trial and the exercise trial (Fig. 3A–D). In premenopausal women, ucOC was not affected, compared to baseline by the glucose load at rest (*P* = 0.16) or following exercise (*P* = 0.46, Fig. 3C).

The associations between glucose levels and BRMs were studied with a multivariable analysis. A higher baseline serum glucose was associated with a lower P1NP (*β *= −0.76, *P* = 0.009), ucOC (*β *= −0.58, *P* = 0.039) and tOC (*β *= −0.51, *P* = 0.10). Insulin was positively associated with P1NP ((*β *= 0.92, *P* = 0.002), tOC (*β *= 0.72, *P* = 0.027) and ucOC (*β *= 0.97, *P* = 0.002). Serum glucose or insulin were not correlated with *β*‐CTX at baseline, but a higher serum glucose post‐OGTT at rest was associated with lower *β*‐CTX levels (*β *= −0.65, *P* = 0.046).

In addition, a higher change in serum glucose following resting OGTT, but not age, BMI or the change in serum insulin, was correlated with greater reduction in bone formation (P1NP, *β *= −0.70, *P* = 0.012 and tOC, *β *= −0.44, *P* = 0.15). The change in the BRMs following OGTT that commenced postexercise did not correlate with the change in either glucose or insulin.

### In vitro

#### Assays of cultured human osteoblasts (HOBs) treated for 2 h with D‐glucose

##### Cell viability

**Figure 4:**
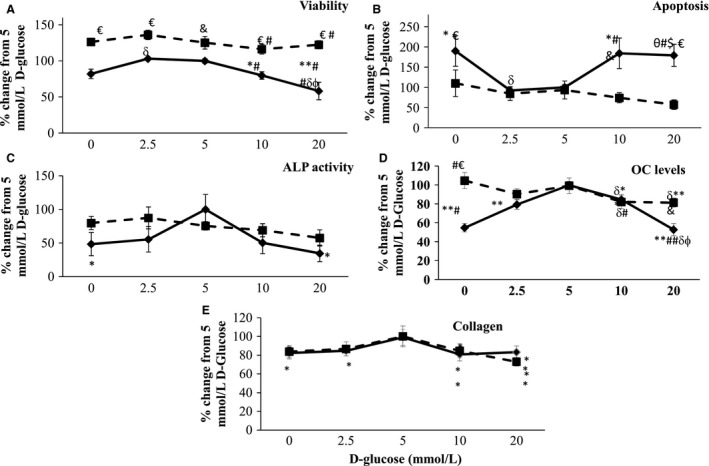
Cell viability (panel A), apoptosis (panel B) ALP and OC activity (panels C and D respectively) and collagen expression (panel E) of cultured human osteoblasts (HOBs) treated with increasing concentrations of D‐glucose. Solid black lines: glucose alone; Dashed lines: glucose plus insulin (50uU mL ^−1^). *P < 0.05 compared to 5 mmol ^−1^, **P < 0.01 compared to 5 mmol ^−1^, #*P *< 0.05 compared to 2.5, ##*P* < 0.01 compared to 2.5, φ*P* < 0.05 compared to 10 mmol L^−1^, δ*P* < 0.05 compared to 0, $*P* = 0.051 between 20 mmol L^−1^ and 5 mmol L^−1^. & *P* < 0.05 between groups, € p < 0.01 between groups.

Glucose (10 and 20 mmol L^−1^) without insulin treatment, reduced cell viability compared with 2.5 and 5 mmol L^−1^ –40%, *P* < 0.05). No glucose (0 mmol L^−1^) reduced cell viability compared with 2.5 and 5 mmol L^−1^. Insulin treatment increased HOBs viability in all D‐glucose concentrations by 25–60% (*P* < 0.05, Fig. 4A).

##### Apoptosis

Caspase 3 activity was lowest at 2.5 and 5 mmol L^−1^ (Fig. 4B). Apoptosis of HOBs was highest at 0, 10 and 20 mmol L^−1^ D‐glucose (*P* < 0.05 compared with 2.5 and 5 mmol L^−1^; Fig. 4B). However, insulin treatment prevented apoptosis at 10 and 20 mmol L^−1^ and in the absence of glucose (*P* < 0.05, Fig. 4B).

##### Alkaline phosphatase

ALP activity increased by twofold (*P* < 0.05) from 0 and 2.5 mmol L^−1^ to 5 mmol L^−1^ D‐glucose (Fig. 4C). ALP was lower in high glucose concentrations (10 mmol L^−1^, *P* = 0.06 and 20 mmol L^−1^, *P* = 0.02 mmol L^−1^) compared with 5 mmol L^−1^ (Fig. 4C). Insulin increase ALP activity at 20 mmol L^−1^ glucose concentrations but not significantly (*P* = 0.11, Fig. 4C).

##### Osteocalcin

RNA expression was highest at 5 mmol L^−1^ glucose alone compared to all other concentrations (*P* < 0.05). The addition of insulin resulted in no change across the glucose concentrations (Fig. 4D).

##### Type 1 collagen

Col1a1 expression was significantly higher at 5 mmol L^−1^ glucose with and without insulin compared to other lower and higher concentrations, however, compared to the expression of other osteoblast markers; this expression seemed relatively stable across all concentrations during this time period.

## Discussion

We have reported, and now confirm, that BRMs are suppressed after a glucose‐load and that higher fasting serum glucose is associated with lower BRMs (Clowes et al. 2002; Iglesias et al. 2011; Schwetz et al. 2014). Glucose‐loading also decreased ucOC in postmenopausal women. Acute exercise did not offset the suppressive effect of the glucose‐load on BRMs. Exposure to high glucose in vitro increased osteoblast apoptosis, reduced osteoblast life span and ALP and osteocalcin expression. Exposure to insulin rescued these deleterious effects on osteoblasts.

Acute high levels of glucose reduce remodeling rate in vivo and osteoblast viability in vitro. It was previously reported that high glucose reduces ALP expression by 50% in osteoblasts and that insulin treatment prevented this effect (Cunha et al. 2014). We extend these observations by demonstrating that high concentrations of glucose suppressed ALP activity and osteocalcin expression in osteoblasts, increased osteoblast apoptosis and so reduced osteoblast survival in vitro; high concentrations of glucose also led to increased Caspase 3 activity, an early marker of apoptotic pathway activation. In addition, these supraphysiological levels of glucose reduced osteoblast viability quantified as the activity of the electron transport chain and the gene expression of markers of bone formation, osteocalcin and type 1 collagen, although the latter was subtle (but significant). While cell culture media often contains high glucose concentrations, we show here that osteoblasts may perform functionally better in more physiological concentrations of glucose. The effects of glucose were not due to osmotic changes as the same concentrations of D‐mannitol had no effect on osteoblasts (data not shown). Importantly, we report that insulin treatment rescued these deleterious effects on osteoblasts in vitro. This in vitro finding is aligned with our in vivo data where higher serum glucose was associated with lower BRMs while higher insulin was associated with higher markers of bone formation and ucOC and a higher serum glucose levels was correlated with a lower *β*‐CTX postresting OGTT.

Thus, long‐term exposure to high glucose levels in patients with T2DM may lead to low bone remodeling which may actually slow bone loss and structural deterioration. However, the less remodeled bone has more time to undergo collagen cross‐linking and more complete secondary mineralization which may compromise these bone ‘qualities’. This may explain the well documented higher risk for fracture despite preservation or increase in BMD in patients with type 2 diabetes (Schwartz et al. 2011; Leslie et al. 2012; Patsch et al. 2013).

**Figure 5:**
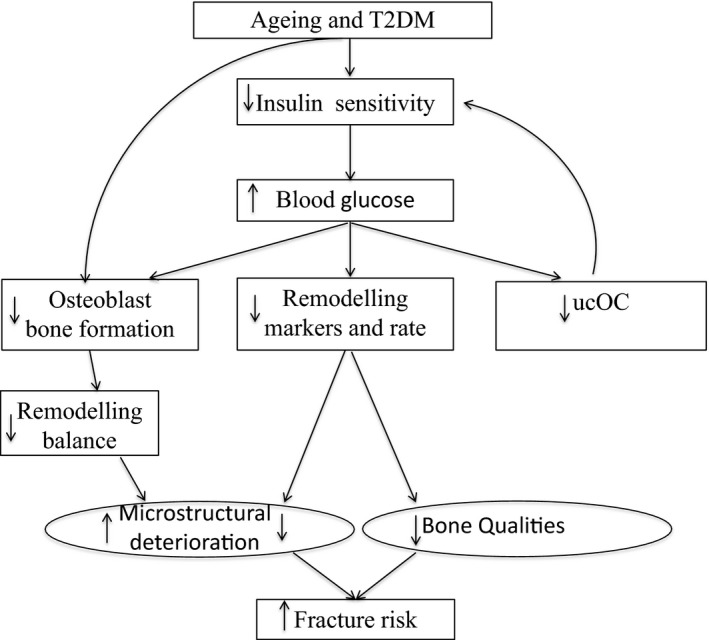
Ageing and T2DM are associated with reduced insulin sensitivity and increased blood glucose concentration which may reduce osteoblast bone formation, remodeling rate and circulating ucOC (undercarboxylated osteocalcin). The reduced ucOC reduces insulin sensitivity. The reduced remodeling rate compromises bone's qualities but slows microstructural deterioration. The reduced osteoblast mediated bone formation reduces remodeling balance increasing microstructural deterioration. The net effect on bone's microstructure and qualities establishes fracture risk.

Following OGTT, with and without exercise, glucose‐loading suppressed BRMs in both groups and ucOC in postmenopausal women. This inability of moderate intensity exercise to prevent the suppressive effect of glucose is not understood, especially considering the high insulin levels observed post‐OGTT following exercise. However, we also observed higher serum glucose levels after the postexercise OGTT (not the resting OGTT) which may interfere with osteoblast function. Nevertheless, our results raise the possibility that the crosstalk between ucOC and glucose metabolism is bidirectional. Conceivably, the low circulating levels of ucOC reported in patients with T2DM as the trigger for higher glucose levels are in fact themselves a result of high circulating levels of glucose. This finding may indicate a feedback loop that regulates both glucose and ucOC as we propose in Figure 5.

The study had several limitations. The sample size was small in the in vivo study. Nevertheless, we did detect suppressive effects of glucose on BRMs in pre and postmenopausal women, but we may have been unable to detect associations between ucOC and BRMs in premenopausal women. The long‐term effects of high glucose levels on bone microarchitecture were not examined. In addition, this study focuses on osteoblasts survival and functions. It is possible that high glucose levels may also have an effect on osteoclasts survival and functions, but this was not assessed in this study and as such future studies should examine the effects of high glucose on these cells. Further work is needed to determine whether exercise failed to modify the suppressive effect of glucose because of the detrimental effect of glucose on osteoblast survival and function.

In conclusion, BRMs and ucOC in women are suppressed after a glucose‐load and acute exercise did not offset the suppressive effect of the glucose‐load on BRMs. This may be, in part, due to increased osteoblast apoptosis, reduced osteoblast life span and ALP activity as well as osteocalcin and collagen RNA expression. Exposure to insulin rescued these deleterious effects on osteoblasts. Future studies should explore whether circulating glucose may be an independent determinant of bone formation at the cellular (BMU) level, and the rate of bone remodeling – the numbers of BMUs remodeling bone at any one time. If so, it is likely to be a net deleterious effect as the reduced volume of bone deposited produces microstructural deterioration while reduced remodeling rate compromises bone matrix composition.

## Conflict of Interest

The authors have nothing to disclose.
